# Cardiac MRI in Fabry disease

**DOI:** 10.3389/fcvm.2022.1075639

**Published:** 2023-02-02

**Authors:** Muhammad Umer, Dinesh K. Kalra

**Affiliations:** Division of Cardiology, University of Louisville, Louisville, KY, United States

**Keywords:** Fabry disease, cardiomyopathy, left ventricular hypertrophy, magnetic resonance imaging, myocardial mapping, feature tracking

## Abstract

Fabry disease is a rare, progressive X-linked inherited disorder of glycosphingolipid metabolism due to a deficiency of α-galactosidase A enzyme. It leads to the accumulation of globotriaosylceramide within lysosomes of multiple organs, predominantly the vascular, renal, cardiac, and nervous systems. Fabry cardiomyopathy is characterized by increased left ventricular wall thickness/mass, functional abnormalities, valvular heart disease, arrhythmias, and heart failure. Early diagnosis and treatment are critical to avoid cardiac or renal complications that can significantly reduce life expectancy in untreated FD. This review will focus on the role of cardiovascular magnetic resonance imaging in the diagnosis, clinical decision-making, and monitoring of treatment efficacy.

## 1. Introduction

Fabry disease (FD) is a rare, monogenic X-chromosome-linked lysosomal storage disorder caused by mutations in the GLA gene. It results in an absence or deficiency of the enzymatic activity of α-galactosidase A (α-GAL) (1). More than 1000 GLA gene variants have been identified–including pathogenic mutations, variants of unknown significance, and benign polymorphisms. The deficiency of α-GAL activity impairs the breakdown of the glycosphingolipid, globotriaosylceramide (GL3)–resulting in progressive accumulation throughout the body, including the blood vessels, heart, kidneys, skin, nervous system, gastrointestinal system, and eyes (2). The massive accumulation of GL3 in cardiomyocytes is detectable as early as childhood and adolescence (3). It activates secondary pathways, including cytokine production, coagulation activation, and oxidative stress (4). GL3-induced oxidative stress in cardiomyocytes causes tyrosine nitration and DNA damage—resulting in contractile dysfunction, myocardial stiffness, and cardiomyocyte apoptosis. GL3 accumulation in the microvasculature causes endothelial injury, intima-media thickening due to smooth muscle cell proliferation, and atheroma production. Cardiomyopathy results from progressive GL3 accumulation in myocytes, valvular fibroblasts, conductive tissue, the microvascular endothelium, and smooth muscle cells. Left ventricular hypertrophy (LVH) is present in 50% of males and 33% of females (1). LVH and diastolic dysfunction occur in the early stages of the disease and eventually progress to systolic dysfunction and heart failure over the next few decades of life.

The prevalence of FD is around 1 in 40,000 to 1 in 117,000 (1) in the general population. However, FD may be more prevalent than previously believed as it is the underlying diagnosis in about 0.5% of patients with non-obstructive hypertrophic cardiomyopathy (prevalence of 1 in 300 in the adult population) (5, 6). Classic FD is defined by absent or very low α-GAL activity (7), early-onset, and progressive multisystemic involvement. In comparison, atypical FD or cardiac variant has some residual or lower than normal α-GAL activity (6), variable onset, and predominantly involves the heart. Heterozygous females may have low-normal or variably deficient α-GAL activity, variable onset, and may develop significant multisystemic manifestations depending on the underlying GLA mutation and X-chromosome inactivation (8, 9). Furthermore, diastolic dysfunction and myocardial fibrosis can develop in females without LVH (10).

## 2. Diagnostic assessment

Fabry disease is a multisystem disease with frequent misdiagnoses and significant diagnostic delays in females (16.3 ± 14⋅7 years) and males (13.7 ± 12⋅9) (9, 11), that adversely affects patient outcomes. FD can significantly reduce life expectancy, by approximately 20 years in males and 15 years in females (12, 13). FD cardiomyopathy includes progressive left ventricular wall thickness, ventricular dysfunction, myocardial ischemia, arrhythmias, and valvular heart disease. Severe microvascular dysfunction is the primary underlying mechanism for myocardial ischemia in the absence of coronary artery disease.

The main diagnostic challenge from a cardiac perspective is distinguishing FD cardiomyopathy from other forms of unexplained LVH, given its infrequent clinical suspicion, especially in the absence of extracardiac manifestations in atypical FD patients and heterozygote females. Increased community awareness will be needed in order to recognize FD as a potential cause of seemingly idiopathic LVH. Confirmation of FD is made by enzyme activity assay and/or genetic testing; tissue biopsy is rarely required. However, genetic testing is the initial screening test in most US centers due to its wide availability. A comprehensive diagnostic approach is needed for early diagnosis and treatment of FD cardiomyopathy, including early recognition of clinical red flags, biomarkers, multimodality cardiac imaging, and assessment for the involvement of other organ systems such as the kidneys, nervous system, etc.

## 3. Cardiovascular magnetic resonance

Cardiovascular magnetic resonance is an essential imaging modality for the quantitative and qualitative assessment of cardiomyopathies. In contrast to transthoracic echocardiography (TTE), CMR provides anatomical and structural evaluation, myocardial strain analysis, and quantitative tissue assessment using late gadolinium enhancement (LGE) and novel parametric mapping techniques like native T1 mapping and extracellular volume (ECV) measurement. It can detect the majority of genotype-positive patients with mild or subclinical cardiac phenotypes. CMR, with advanced mapping techniques, is a valuable diagnostic tool in asymptomatic carriers and preclinical deposition of GL3 in the myocardium, microvasculature, conduction system, and valves (14). Multiparametric CMR, along with biomarker testing, picks up the majority of cases of early organ involvement in mild FD (15). This significantly impacts decision-making in asymptomatic disease, as current guidelines recommend treatment when imaging features indicate myocardial involvement. However, CMR is less readily available than TTE and requires an experienced technician and interpreter, and medical device incompatibility or artifacts may limit accurate evaluation.

### 3.1. Structural evaluation

Cardiovascular magnetic resonance is the gold standard for the assessment of myocardial wall thickness and mass. In comparison, TTE is limited by acoustic windows, overestimating or underestimating wall thickness and mass, dropout artifacts in the basal inferolateral wall and RV myocardium, and lower reproducibility (17).

#### 3.1.1. Left ventricle

Left ventricular hypertrophy is the most common structural change reported in FD (18). Patients predominantly have concentric LVH at the beginning (1). Asymmetrical hypertrophy with a grossly thickened septum compared to the inferolateral wall develops in late stages–replacement fibrosis causing wall thinning of the latter. Kampmann et al. (19) noted that the severity of LVH progresses with age, occurring 10–15 years later in females than in males. Females are less likely to develop LVH than males (33% vs. 50%) (1). Left ventricular mass (LVM) is directly related to left atrial thickness and dimensions. CMR analysis is also valuable due to the higher contribution of papillary muscles and trabeculations to total LVM in FD patients (20, 21).

#### 3.1.2. Right ventricle

Right-sided structural changes are common in FD, typically right ventricular hypertrophy (RVH), with preserved systolic function and normal chamber size. However, diastolic dysfunction often exists that may progress to advanced heart failure (22). Niemann et al. (23) noted that RVH was evident in 71% of the patients at baseline. A significant positive correlation existed between left and right ventricular wall thickness. ERT showed no beneficial effects on RV morphology and function in this study. However, in another study by Wuest et al. (24), ERT significantly reduced RV mass (baseline 31 ± 6 g/m^2^ vs. follow-up 27 ± 7 g/m^2^, *p* < 0.05).

### 3.2. Functional evaluation

Fabry disease is different from other interstitial cardiomyopathies–GL3 accumulation is intracellular, resulting in a true increase in LV myocyte mass and a reactive LVH. It impairs ventricular compliance, increases filling pressures, and restricts diastolic filling, causing heart failure (19). CMR is highly accurate and reproducible in measuring ejection fraction (EF) and ventricular volumes and does not rely on geometric assumptions as in TTE. It can determine small changes in ventricular function and volume on serial assessment and is especially helpful in quantifying the impact of therapy.

#### 3.2.1. Myocardial strain analysis

Cardiovascular magnetic resonance measurement of myocardial deformation and mechanics by strain and strain rate analysis is an emerging tool for the quantitative assessment of global and regional cardiac function in cardiomyopathies, often providing a preclinical diagnosis. Feature tracking-CMR (FT-CMR) is a very feasible and highly accurate technique for strain/strain rate analysis in cardiac diseases, especially the assessment of LV-GLS (global longitudinal strain) in LVH has excellent reproducibility (25). It is more accurate in assessing all myocardial segments and independent of intramyocardial features compared to TTE-speckle tracking. Mathur et al. (26) demonstrated the reproducibility of CMR strain abnormalities in FD. Base-to-apex circumferential strain (CS) gradient was lower in FD patients compared to healthy controls (2.1 ± 3.7% vs. 6.5 ± 2.2%, *p* = 0.002), and it was able to discriminate between FD patients without LVH or LGE from healthy controls, endorsing it as an early marker of cardiac involvement in FD. In a study by Roller et al. (16), GLS was significantly reduced in FD patients (*p* = 0.0009) and correlated with Lyso-GL3 elevation. GLS values increased with worsening LVH and LGE. Another study by Vijapurapu et al. (27) demonstrated that in LVH-negative FD patients, GLS impairment was correlated with a reduction in T1, suggesting that mechanical dysfunction occurs before GL3 accumulation. In conclusion, FT-CMR abnormalities are reproducible imaging biomarkers for early cardiac involvement in FD.

### 3.3. Tissue characterization

#### 3.3.1. Late gadolinium enhancement

**FIGURE 1:**
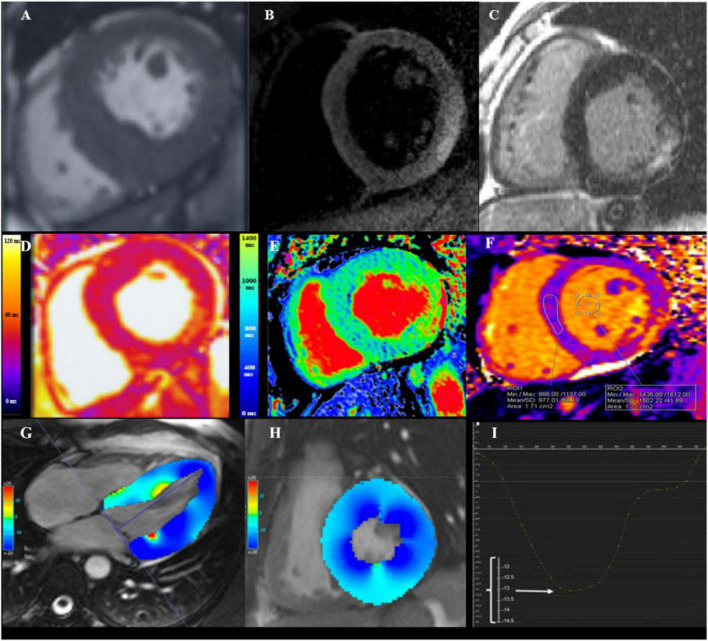
Cardiovascular magnetic resonance (CMR) assessment in Fabry disease (FD). **(A)** Steady state free precession (SSFP) CINE short-axis view showing increased wall thickness of mid-inferoseptum measuring 22 mm. **(B)** Dark-blood T2 short inversion-time, inversion-recovery (STIR) image showing myocardial edema (arrowheads) in the basal inferolateral wall (BIFL). **(C)** Late gadolinium enhancement (LGE) imaging showing mid-myocardium BIFL LGE in short-axis view. **(D)** Native T2 mapping showing high myocardial T2 value in BIFL (54 ms; normal reference value 45 ± 2 ms for this 1.5 T scanner). **(E)** Native T1 mapping showing low myocardial T1 value in the septum (812 ms; normal reference value 984 ± 18 ms for this 1.5 T scanner). **(F)** Native T1 mapping in advance disease showing pseudonormalization of T1 value in the septum and increased T1 in the BIFL. **(G)** Long-axis CINE SSFP image with color-coded myocardial longitudinal strain map. **(H)** Short-axis CINE SSFP image with color-coded myocardial circumferential strain map. **(I)** Decreased global longitudinal strain of −13.2%, as the enlarged scale on the *Y*-axis showed [adapted from Roller et al. (16)].

Late gadolinium enhancement reflects replacement fibrosis and helps differentiate FD cardiomyopathy from ischemic and other hypertrophic cardiomyopathies. LGE is present in almost half of FD patients and typically involves the basal and mid inferolateral myocardium in about 75% of these patients (28, 29) (Figure 1). About one-fourth of FD females can develop LGE without LVH (10). TTE can miss nearly half of the early-stage cardiomyopathy cases in females; however, the majority of these will be detected by CMR. Thus, the assessment of fibrosis by CMR is crucial in the screening and staging of FD, especially in female patients who may not meet conventional LVH criteria early on by TTE (10). Liu et al. (30) studied the association between diastolic dysfunction and myocardial fibrosis in FD. LGE was present in 38% of FD patients, mostly at the basal and mid-segments of the inferolateral wall. In 9% of patients, LGE was present without functional abnormalities. This indicates that LGE can be present in FD patients with normal diastolic and systolic function; thus, chronic inflammation likely contributes to the development of replacement fibrosis.

#### 3.3.2. T1 mapping

Standard imaging evaluation of anatomical and functional abnormalities and the presence of replacement fibrosis lack sensitivity or specificity to diagnose FD. Quantifying longitudinal relaxation time (T1) generates a pixel-wise color-encoded map of the myocardium, allowing the detection of very subtle pathological changes at the microscopic level that may be indicative of the preclinical stage. Native myocardial T1 values, obtained without a paramagnetic contrast agent, are higher in fibrosis, edema, and amyloid but lower in iron overload and focal fat infiltration. Accumulation of sphingolipids inside lysosomes in FD significantly shortens the native T1 values. T1 mapping has overcome the limitations of gadolinium contrast agent used in patients with advanced renal disease. In addition, T1-mapping is superior to LGE, with higher diagnostic accuracy, when the myocardium has more uniform and diffuse involvement (31).

Pica et al. (32) found T1 mapping highly reproducible in FD patients. It had 48% sensitivity and 99% specificity in distinguishing LVH-negative FD subjects from healthy volunteers. Reduced native T1 was highly prevalent (89%) in LVH-positive FD patients. LVH-negative FD patients had a 48% prevalence of reduced native myocardial T1, which was associated with advanced echocardiographic parameters of cardiac dysfunction (GLS and E/e’ ratio). In FD, native T1 values are reduced in the early stages but begin to normalize with progressive GL3 accumulation. T1 values are increased in the advanced stages due to replacement fibrosis and ongoing inflammation.

In patients with LVH, T1 mapping could differentiate FD from other phenotypes. In a study by Sado et al. (33), FD patients had lower septal T1 values (FD vs. healthy volunteers vs. other patients; 882 ± 47, 968 ± 32, 1018 ± 74 ms, *P* < 0.0001), which were inversely related to LV wall thickness (*r* = -0.51; *P* = 0.0004). In 40% of the FD patients without LVH, T1 values were abnormal due to the early phase of GL3 accumulation in myocytes.

Thompson et al. (34) compared LV mass, wall thickness, mass/volume ratio, LVEF, myocardial T1 values, and ECV as potential disease-specific imaging biomarkers of FD. The study concluded that reduced native myocardial T1 values are the most sensitive and specific CMR parameter in FD patients, irrespective of sex, LV morphology, or function. Native myocardial T1 values were substantially lower in FD (1070 ± 50 ms) as compared to healthy controls (1177 ± 27 ms) and concentric remodeling or hypertrophy (1207 ± 33 ms). Pagano et al. (35) evaluated RV myocardium by T1 mapping in FD, pulmonary hypertension (PH), and healthy controls. FD patients with thickened RVs had similarly reduced native T1 values in the RV and LV. This was the first report of reduced native T1 values in the RV. PH patients with thickened RVs showed increased native T1 values in both ventricles suggesting fibrosis. However, T1 mapping of the RV remains challenging due to relative thinness and the possibility of contamination from the blood pool or epicardial fat.

T1 mapping parameters, as surrogates for GL3 accumulation, can be reduced in the absence of LVH and basal inferolateral wall (BIFL) LGE. Thus, it may play a potential role in detecting the most appropriate patients for treatment and early quantitative assessment of treatment efficacy.

#### 3.3.3. T2 mapping

T2 mapping sequences measure T2 relaxation times representing myocardial edema/inflammation (36). Nordin et al. (37) demonstrated elevated native T2 values in the early stage of myocardial involvement, later corresponding to areas of LGE in the BIFL. High-sensitivity troponin T was elevated in 40% of the patients (75th percentile: 32 ng/l; range 3–93 ng/l; normal reference 0–14 ng/l), and increased T2 value was the strongest predictor (*B* = 2.4; *p* < 0.001). All patients with elevated troponin had LGE representing inflammation instead of scar. Chronic T2 elevation in LGE areas and elevations of global T2 values are both associated with poor outcomes (38). These findings suggest FD as inflammatory and infiltrative cardiomyopathy.

#### 3.3.4. Extracellular volume

Myocardial ECV is calculated from native and contrast enhanced T1 values of myocardium and blood as well as patient’s hematocrit. ECV, a measurement of the size of the extracellular space, is elevated in amyloidosis and other infiltrative diseases, but in their absence, it is a biomarker for myocardial fibrosis. ECV values are normal in FD as it is an intracellular storage disease (39). However, as cardiomyopathy progresses, ECV values may increase in the areas of myocardial fibrosis (37). Hypertrophic cardiomyopathy (HCM) has increased ECV values due to extracellular matrix expansion and myocardial disarray, whereas ECV is reduced in athlete’s heart due to an increase in healthy myocardium by cellular hypertrophy.

### 3.4. Myocardial perfusion

Fabry disease patients frequently experience angina, and microvascular dysfunction is the primary underlying mechanism correlating with the extent of replacement fibrosis (40). CMR perfusion mapping provides a rapid quantitative assessment of microvascular dysfunction. Knott et al. (41) demonstrated that FD patients had lower stress myocardial blood flow maps (MBF) than healthy controls, even in the absence of LVH. MBF decline, especially in the endocardium, correlates with disease severity and can be an early disease marker.

### 3.5. Pediatric population

Society for cardiovascular magnetic resonance (SCMR) guidelines do not provide FD-specific recommendations for CMR evaluation in the pediatric population (42). In young athletes, CMR is the preferred imaging modality to differentiate LVH from physiological remodeling by assessing hypertrophy regression with deconditioning (43). CMR provides more accurate wall thickness measurements and LVM compared to TTE (44). FT-CMR and T1 mapping techniques can identify myocardial fibrosis without using contrast agents (45). CMR use is limited in children due to a higher risk of anesthesia, the lower signal-to-noise ratio in small children, reduced temporal resolution due to higher heart rates, and difficulty holding breath under anesthesia.

## 4. Differentiation of hypertrophic myocardium, patient selection and quantitative assessment of treatment efficacy

Multiparametric CMR has a vital role in differentiating FD cardiomyopathy from other etiologies of LVH and can strongly impact clinical decision-making and prognosis by initiating disease-specific therapies (46) (Table 1). Cardiac and renal disease may not manifest clinically until adolescence or adulthood. Furthermore, renal damage is typically subclinical in early stages and requires biopsy for identification. Children with FD mutations should be treated as soon as the symptoms develop. Although in asymptomatic boys with classic FD mutation, treatment should be considered as early as 8–10 years of age (47). To avoid potentially irreversible complications, CMR is essential in early recognition and clinical decision-making. The European Fabry Working Group consensus statement recommends initiation of therapy in both classic and non-classic FD patients of both sexes when there is an increased LV wall thickening >12 mm (Class 1 recommendation) (48). However, major cardiology guidelines do not provide FD-specific recommendations for CMR. Nonetheless, it is vital to further study the potential role of strain analysis and T1/T2 mapping in treatment initiation and as an early quantitative measure of its efficacy. LVM reduction varies among various studies (10–27%), likely depending on the timing of therapy, the intensity of therapy, stage of cardiomyopathy, and other confounding factors such as age, sex, hypertension, etc. (Table 1).

**TABLE 1:** Role of CMR parameters in characterizing the etiology of hypertrophic cardiomyopathy and the quantitative assessment of treatment efficacy in Fabry disease (FD).

	LVH pattern	LGE pattern	T1 mapping	T2 mapping	Extracellular volume (ECV)	Strain analysis
Fabry disease	Majority of the patients have concentric LV wall thickening (49).	Basal to mid inferolateral mid-myocardium (29).	Initially native T1 values are reduced but later there is pseudonormalization in the areas of LGE. It can reliably distinguish FD from other causes of LVH (27).	T2 values are elevated in the area of LGE, indicating chronic inflammation (37, 50).	ECV is normal (39), but may increase in the area of LGE as a biomarker for fibrosis.	GLS and GRS are significantly reduced, and GLS impairment correlates with GL3/Lyso-GL3 elevation, thus having a potential role in detecting early cardiac involvement (16, 27). Loss of base-to-apex CS gradient is also an early marker of cardiac involvement (26).
Hypertrophic cardiomyopathy (HCM)	Asymmetric and involves anteroseptal wall in 70% of the cases. (51) Variants include apical and mid-ventricular.	Patchy involvement in the areas of hypertrophy.	Patchy areas of elevated native T1 values in hypertrophied myocardium, even in the absence of LGE (52).	Elevated T2 values indicate areas of active tissue injury (53).	ECV is elevated in hypertrophied myocardium (52) and correlates with the percentage of LGE (39).	GLS of ≤−12.8% and SLS of <−12.5% have high diagnostic accuracy for patchy fibrosis (45, 54) in HCM.
Hypertensive heart disease (HHD)	Concentric LV wall thickening with asymmetric basal septum involvement (49, 55).	No significant or specific pattern of LGE.	Normal native T1 values.	Normal T2 values.	ECV is normal.	GLS is significantly lower and can help differentiate from other LVH phenotypes. Diagnostic accuracy is similar to global native T1 and LGE (56).
Aortic stenosis (AS)	LV wall thickness can be normal or have concentric remodeling, symmetric hypertrophy, or eccentric wall thickening (41). The degree of LVH is an independent predictor of higher cardiovascular events (57).	No significant pattern. LGE may be present at RV insertion points (58).	High native T1 values are an independent predictor of adverse outcome (59).	Significantly elevated T2 in severe AS shows a potential role of inflammation in myocardial remodeling (60).	Elevated ECV is a stronger predictor of adverse cardiovascular outcomes than the extent of LVH and is a powerful independent predictor of mortality (61).	FT-CMR longitudinal strain/velocity is significantly reduced in severe AS and strongly correlates with hemodynamic sub-grouping (62).
Cardiac amyloidosis (CA)	Concentric LV wall thickening (49).	Diffuse LV transmural or sub-endocardial LGE (63). (ATTR > AL) (64) Atrial wall and RV free wall may also have diffuse LGE.	Elevated native T1 values. (AL > ATTR) (65) T1 mapping and ECV have superior diagnostic values compared to strain analysis (66).	Elevated T2 is due to myocardial edema caused by the toxic effect of amyloid deposition on cardiomyocytes and is a predictor of prognosis (67).	Significantly elevated ECV values (ATTR > AL) (68).	GLS is significantly reduced in CA compared to FD and HCM, with “relative apical sparing” (69).
Quantitative assessment of treatment efficacy in FD	Structural and functional parameters			Late gadolinium enhancement	Parametric mapping techniques	
	Weidemann et al. (70) noted a statistically significant 28% decrease in LV inferolateral wall thickness and 10% decrease in LV mass by CMR in patients treated with ERT for 12 months. Peak systolic strain rate and end-systolic strain increased significantly in the posterior wall also. Both radial and longitudinal strain showed improvement. Hughes et al. (71) followed FD patients after treatment with agalsidase-α by CMR and TTE and noted regression of LVH due to progressive clearance of GL3 content from cardiomyocytes. Nordin et al. (50) demonstrated that after 12 months of ERT, LVH-positive patients had a detectable, small reduction in LVMi (117 ± 38 versus 114 ± 36 g/m^2^; *P* = 0.048). There was no significant change in GLS in both LVH-positive and LVH-negative groups. Koeppe et al. (72) observed a significant decrease in end-diastolic wall thickness and a decline in hypokinesia after 12 months of ERT in LGE-negative patients. Wuest et al. (24) followed FD patients for 13 ± 1 months after ERT; there was a significant reduction in LV and RV mass, LV and RV EDV and LV ESV, while LVEF increased significantly. There was no significant change in RV ESV, SV, and EF. Imbriaco et al. (73) evaluated FD patients after 45 months of ERT agalsidase-β; LV mass and LV wall thickness reduced significantly. There was no significant change in LVEF. Messalli et al. (74) evaluated FD patients with CMR after 48 months of treatment with agalsidase-β, and a significant reduction in LV mass and wall thickness was observed. There was no significant change in LVEF.			No significant change was noted.	Nordin et al. (50) demonstrated that after 12 months of ERT, LVH-positive patients had a detectable, small reduction in native T1 lowering (partial normalization; 920 ± 48 ms vs. 902 ± 47; *P* = 0.008). However, in LVH-negative patients, who were all females, the reduction in native T1 lowering was not statistically significant (940 ± 46 vs. 948 ± 60 ms; *P* = 0.480). Overall, 83% had an increase in native T1 value after 1 year of ERT. There was no significant change in ECV in both LVH-positive and LVH-negative groups. Further research will be required to compare long-term clinical outcomes and prognosis in patients with native T1 normalization vs. patients with no change or native T1 reduction with ERT. Imbriaco et al. (73) evaluated FD patients after 45 months of ERT agalsidase-β, and a significant reduction in native T2 values was noted in all myocardial regions. Messalli et al. (74) observed a significant reduction in native T2 values after 48 months of ERT with agalsidase-β.	

TTE, transthoracic echocardiography; CMR, cardiovascular magnetic resonance imaging; LVH, left ventricular hypertrophy; LA, left atrium; LVMi, left ventricular mass index; EF, ejection fraction, EDV, end-diastolic volume, ESV, end-systolic volume; SV, stroke volume; LGE, late gadolinium enhancement; FT-CMR, feature tracking cardiac magnetic resonance imaging; GLS, global longitudinal strain; CS, circumferential strain; SLS, segmental longitudinal strain; GRS, global radial strain; AL, light-chain amyloidosis; ATTR, transthyretin amyloidosis.

## 5. Treatment options

An interdisciplinary FD center should perform therapy planning and initiation. The main therapeutic goals are symptom reduction to improve quality of life and preventing or halting multiorgan involvement to improve life expectancy. Established treatment options to reduce GL3 accumulation include replacing deficient endogenous α-GAL with recombinant enzyme replacement therapy (ERT) or increasing α-GAL enzyme activity inside lysosomes by chaperone therapy. Current ERT options include intravenous agalsidase-α (71) or agalsidase-β (73, 74). Oral chaperone therapy with migalastat corrects the misfolding of α-GAL and increases its intra-lysosomal availability. Next-generation plant-derived forms of ERT include pegunigalsidase-α (75) and moss α-GAL (76) with increased plasma half-life and reduced immunogenicity. Other emerging therapies include substrate reduction and gene therapy. Substrate reduction therapy aims to decrease the substrate concentration and subsequently inhibit GL3 accumulation in the cells. Lucerastat (77) and venglustat (78, 79) inhibit glucosylceramide synthase (GCS) to reduce the biosynthesis of glucosylceramide (GL1) and downstream GL3. Gene therapies are being developed as a long-term treatment option to cause endogenous α-GAL expression within disease phenotype cells, including α-GAL cDNA insertion *via* lentivirus (80), adeno-associated virus (AAV) gene delivery (NCT04455230) and gene-editing technology such as CRISPR (clustered regularly interspaced palindromic repeats)/Cas (CRISPR-associated genes).

## 6. Prognosis

Cardiomyopathy is the leading cause of death in men (34%) and women (57%) with FD (81). Early diagnosis is vital to prevent cardiac involvement and stop disease progression to avoid life-threatening complications of arrhythmias, myocardial infarction, and heart failure. The efficacy of treatment decreases with advancing stages of cardiomyopathy (2), thus worsening the overall prognosis. Orsborne et al. (82) developed a prognostic model based on age, native myocardial T_1_ dispersion, and left ventricular mass index (LVMi) to provide an accurate estimate of the 5-year risk of adverse cardiac outcomes. CMR-derived myocardial T_1_ relaxation time with wider distribution may have a greater prognostic value as it can better reflect GL3 accumulation, fibrosis/inflammation, and thus disease severity. LVMi by CMR is independently associated with adverse cardiac events in FD (83). Other clinical indices of organ involvement such as renal function, proteinuria, and neurological dysfunction also portend long-term prognosis.

## 7. Conclusion

Cardiac involvement should be detected promptly in FD patients to prevent disease progression and life-threatening complications. Multiparametric CMR imaging can play a vital role in reaching the correct diagnosis of hypertrophic myocardium and differentiating it from other phenotypes. FT-CMR and parametric mapping are emerging techniques with the potential for preclinical detection of cardiac involvement and monitoring response to therapy. In particular, T1 mapping is a superior technique for detecting GL3 accumulation and diffuse fibrosis. It has the potential for quantitative assessment of treatment efficacy–current data is insufficient, and further research is required to establish this role.

## Author contributions

Both authors contributed to review conception, design, research, writing, and editing and approved the submitted version.
